# Consequences of biodiversity loss diverge from expectation due to post-extinction compensatory responses

**DOI:** 10.1038/srep43695

**Published:** 2017-03-03

**Authors:** Matthias S. Thomsen, Clement Garcia, Stefan G. Bolam, Ruth Parker, Jasmin A. Godbold, Martin Solan

**Affiliations:** 1 Ocean and Earth Science, National Oceanography Centre Southampton, University of Southampton Waterfront Campus, European Way, Southampton. SO14 3ZH, UK; 2 Cefas Laboratory, Pakefield Road, Lowestoft, Suffolk, NR33 0HT, UK; 3 Biological Sciences, University of Southampton, Highfield Campus, Southampton. SO17 1BJ, UK

## Abstract

Consensus has been reached that global biodiversity loss impairs ecosystem functioning and the sustainability of services beneficial to humanity. However, the ecosystem consequences of extinction in natural communities are moderated by compensatory species dynamics, yet these processes are rarely accounted for in impact assessments and seldom considered in conservation programmes. Here, we use marine invertebrate communities to parameterise numerical models of sediment bioturbation – a key mediator of biogeochemical cycling – to determine whether post-extinction compensatory mechanisms alter biodiversity-ecosystem function relations following non-random extinctions. We find that compensatory dynamics lead to trajectories of sediment mixing that diverge from those without compensation, and that the form, magnitude and variance of each probabilistic distribution is highly influenced by the type of compensation and the functional composition of surviving species. Our findings indicate that the generalized biodiversity-function relation curve, as derived from multiple empirical investigations of random species loss, is unlikely to yield representative predictions for ecosystem properties in natural systems because the influence of post-extinction community dynamics are under-represented. Recognition of this problem is fundamental to management and conservation efforts, and will be necessary to ensure future plans and adaptation strategies minimize the adverse impacts of the biodiversity crisis.

Species extinction and the associated degradation of ecosystems are proceeding at an accelerating pace^1^,^2^,^3^, but the consequences of the current decline in biodiversity for socio-ecological systems represent a critical knowledge gap for policy-makers^4^. Consensus reached from experiments that have manipulated biodiversity and measured various ecosystem functions, including primary productivity, nutrient cycling and decomposition, predict an accelerating decline in ecosystem properties with increasing biodiversity loss^5^, yet it is not clear how appropriate it is to apply this general relationship at the landscape scale^6^. A major difficulty is that the complexities of natural communities^7^,^8^, including the role of rare species^9^ and the occurrence of co-extinctions^10^, have been poorly articulated in controlled experiments, and very few studies have focussed on realistic trajectories of species loss that factor in a predisposition to community dynamics^11^. Indeed, the role of population dynamics in moderating the consequences of extinction have received little attention^12^,^13^,^14^, despite direct evidence that communities undergo fundamental shifts in the relative abundance of taxa and the dominance of species in each successive assemblage that follows an extinction event^15^,^16^. Such community turnover resembles that of recovering post-disturbance communities^17^, where a variety of compensatory interactions amongst surviving species^18^ develop and offset, wholly or in part, the functional contributions made by species that have been extirpated^14^.

Compensatory responses tend to be asynchronous within a perturbed community and can lead to to partial^19^,^20^, complete^21^,^22^,^23^, or over^24^ compensation in ecosystem functioning. Species can also switch behaviour^25^, make physiological regulation adjustments^26^, exhibit elevated growth (biomass compensation^27^) or increase reproductive investment (numeric compensation^28^) in response to perturbation, especially following release from competition/predation^29^ or during niche expansion^30^. These responses may not be immediate, in some instances taking months^31^ to years^32^ to develop, but once expressed they can be critical in influencing further species interactions^33^ and can dramatically affect ecosystem properties^34^,^35^,^36^,^37^. Furthermore, where extinction events are localized and communities are interconnected^38^, immigration and re-colonization from the surrounding area^39^, as well as recruitment from the wider species pool^40^, can play a pivotal role in stabilizing local population decline and ecological processes^41^. Ultimately, however, the degree of functional compensation will depend on the amount of functional redundancy in the surviving community, which, in turn, will reflect the spatial extent and consistency of the perturbation compromising biodiversity and the level of covariation between the extinction driver and the traits that mediate functioning^42^.

Despite the range of compensatory mechanisms and variety of ways in which species interactions can affect the functional properties of natural communities^7^,^18^, few studies have explored how surviving species moderate the consequences of extinction^42^,^43^,^44^,^45^ and the relative role of different compensation mechanisms remain largely unexplored. Here, we use probabilistic numerical simulations to test how the loss of sediment dwelling marine invertebrates may affect the sediment mixing depth, an important mediator of biogeochemical cycling^46^,^47^. Our simulations assume that the sequence of species loss is either random or ordered by body size or rarity to reflect likely sources of extinction risk. We compare these probabilistic distributions to further simulations in which populations of surviving species maintain total abundance (numeric compensation) or total biomass (biomass compensation) sourced from different components (common or rare species, within or between bioturbation functional groupings, same or different/lower or higher level of activity) of the surviving community. In doing so, our objective is to establish the extent to which alternative compensatory dynamics alter biodiversity-function relationships.

## Results

**Figure 1:**
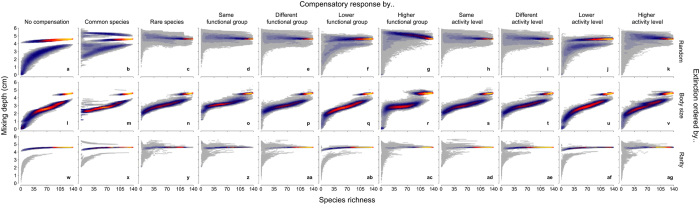
Predicted changes in sediment mixing depth following extinction of sediment-dwelling invertebrates and post-extinction numeric compensation. Simulations (n = 1000 per panel) represent species losses that occur at random (panels a–k) or which reflect trait-based vulnerabilities to extinction governed by body size (panels l–v) or rarity (panels w–ag). We assumed that the surviving community shows either no compensatory response (a,l,w) or full numeric compensation by common (b,m,x) or rare (c,n,r) species, species from within (d,o,z), between (e,p,aa), lower (f,q,ab) or higher (g,r,ac) functional groups, or species with the same (h,s,ad), different (i,t,ae), lower (j,u,af) or higher (k,v,ag) level of activity to the species that have gone extinct. Colour intensity (cold to warm colouration; grey - blue – red - yellow) reflects an increasing density (low to high) of data points.

**Figure 2:**
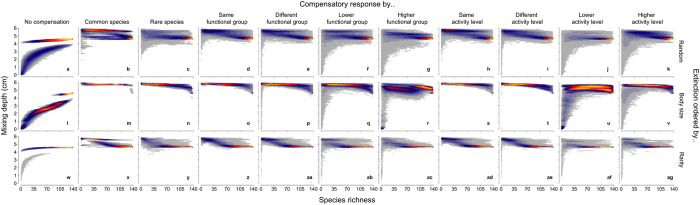
Predicted changes in sediment mixing depth following extinction of sediment-dwelling invertebrates and post-extinction biomass compensation. Simulations (n = 1000 per panel) represent species losses that occur at random (panels a–k) or which reflect trait-based vulnerabilities to extinction governed by body size (panels l–v) or rarity (panels w–ag). We assumed that the surviving community shows either no compensatory response (a,l,w) or full biomass compensation by common (b,m,x) or rare (c,n,r) species, species from within (d,o,z), between (e,p,aa), lower (f,q,ab) or higher (g,r,ac) functional groups, or species with the same (h,s,ad), different (i,t,ae), lower (j,u,af) or higher (k,v,ag) level of activity to the species that have gone extinct. Colour intensity (cold to warm colouration; grey - blue - red - yellow) reflects an increasing density (low to high) of data points.

In the absence of compensatory dynamics, the form of the biodiversity function curve approximates expectations (accelerating loss of function with declining species richness, moderated by how extinctions are ordered), and our simulations include a previously documented^42^ prominent bifurcation of the mixing depth that reflects whether a burrowing brittlestar, *Amphiura filiformis*, is present (deeper mixing depth) or absent (shallower mixing depth) in the surviving community (panels a,l,w in Figs 1 and 2). The disproportionate contribution of this species is evident throughout all of our simulations (Supplementary Figures S1 and S2), although it is clear that the loss of other species can also result in stepped changes (abrupt shifts in mixing depth, most prominently featured in Fig. 1) that show a tendency to only partially compensate for the loss of the extirpated species (Fig. 1).

**Figure 3:**
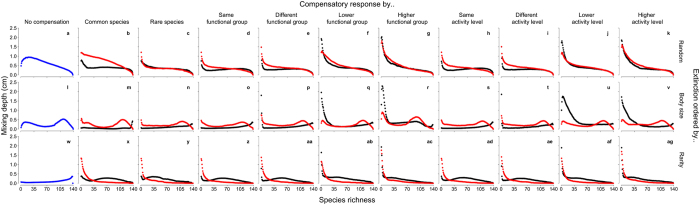
Variability of sediment mixing depth following extinction of sediment-dwelling invertebrates and post-extinction compensation. Standard deviations are shown for the probabilistic distributions (n = 1000) at each level of species richness for the extinction scenarios and compensatory responses depicted in Figs 1 and 2, assuming no compensatory response (blue), full numeric compensation (red) or full biomass compensation (black).

When compensatory mechanisms of the surviving community are based on numerical responses, the mixing depth is largely maintained as species richness declines (Fig. 1), when extinctions are random (panels b–k) or ordered by rarity (panels x–ag). However, when extinctions are ordered by body size (panels m–v), compensatory responses, independent of how they are expressed, are unable to fully mitigate the functional consequences associated with species loss, yet notable differences exist depending on which species drive the compensatory response. However, when compensatory mechanisms of the surviving community are based on biomass, with a few exceptions, there is a tendency for overcompensation to take place along the main species-function trajectory (colour intensity in Fig. 2). Despite implementing these fundamentally different compensatory mechanisms, our simulations reveal that most probabilistic trajectories overlap one another to form a narrow band of likely ecosystem functioning (colour intensity in Figs 1 and 2). The variation of the outcome under numeric versus biomass compensation increases at low and high levels of species richness; this is particularly evident for compensatory responses driven by lower and higher functional groupings and activity level, but at intermediate levels of species richness there is some evidence to suggest similar levels of mixing depth, irrespective of the type of compensation mechanism (Fig. 3). The same general patterns persist in the absence of *A. filiformis*, although variation is considerably reduced for numeric compensation (Supplementary Figure S3).

When compensation is realized via the most common species in the surviving community, our models indicate that numeric compensatory mechanisms (Fig. 1, panels b,m,x) can reduce, maintain, or increase the mixing depth relative to when there is no compensation, whilst biomass compensatory mechanisms (Fig. 2, panels b,m,x) show a tendency to increase the mixing depth. In contrast, when compensation is realized by the rarest species in the surviving community (panels c,n,y in Figs 1 and 2), the mixing depth tends to increase relative to the no compensation scenarios under both numeric and biomass compensation, albeit with high variability at low levels of species richness (panels c,n,y in Fig. 3). When compensating species stem from the same or different functional group, or exhibit identical or contrasting levels of activity relative to the species that have been extirpated (compare panels d-e, o-p, z-aa and h-i, s-t, ad-ae between Figs 1 and 2), there is little influence of compensation because compensation is sourced from all possible functional groups. Hence, over multiple species losses, compensation does not occur disproportionately in any one functional group. When compensation is directed towards lower functional groups (Figs 1f,q,ab and 2f,q,ab) and/or activity levels (Figs 1j,u,af and 2j,u,af), the loss of functioning accelerates relative to the other functional group and/or activity level scenarios because bioturbation capacity is reduced. Alternatively, when compensation is directed towards higher functional groups (Figs 1g,r,ac and 2g,r,ac) or activity levels (Figs 1k,v,ag and 2k,v,ag), the mixing depth tends to be maintained at similar or higher levels than those observed when compensation stems from species with the same or different functional group/activity level because bioturbation capacity continues to be maintained or is increased. These observations suggest that certain traits may well be linked to the mediation of ecosystem functioning, but their role in determining observed levels of functioning will depend on how influential such traits are for underlying ecosystem processes^48^.

## Discussion

Using numerical models parameterised with data from a marine benthic community, we have demonstrated that incorporation of compensatory dynamics, irrespective of the mechanism of compensation (numeric or biomass) or how a species extinction risk is determined (random, ordered by rarity or body size), have the potential to lead to clear differences in aggregate community responses to species loss that do not always conform to expectations based on the generalised biodiversity-function relation curve^5^. Our models showed that the probabilistic distributions for sediment mixing depth were deeper when compensatory mechanisms were present relative to when they were absent, and when compensatory dynamics reflected post-extinction increases in biomass rather than abundance. Moreover, simulations indicate that the way in which numeric or biomass compensation is expressed within the surviving species pool is most influential. These observations highlight the importance of post-extinction compensatory mechanisms in determining how traits are expressed and mediate function, and suggest that the ecological status of soft-sediment benthic habitats is unlikely to conform to expectation as current visions of future scenarios of extinction lack the necessary sophistication.

Numerous studies investigating the consequences of environmental forcing for ecosystem functioning have focused on the decline of the number of species and their functional traits or attributes. Whilst the effect of various attributes of biodiversity on ecosystem functioning, including evenness^49^, dominance^50^, and functional traits^51^ have been studied, the relative importance of compensatory mechanisms in natural ecosystems and the concept of compensation as a whole has largely been ignored despite evidence for the occurrence of compensation in natural systems^52^. Importantly, our simulations reveal that the mixing depth of sediment-dwelling invertebrate communities will depend on how compensatory behaviour is expressed, and the extent to which the functional attributes of compensating species affect bioturbation. A difficulty with determining the latter is that recent work has shown that differences in how species interact with sediment biogeochemistry and other aspects of the environment can converge in terms of absolute effects of ecosystem properties^48^,^51^. This may explain previous inconsistencies in linking particular species traits to ecosystem functioning^47^ and why community compensation does not appear to be dominated by a limited number of species that host specific sets of traits; a conclusion consistent with recent studies that demonstrate the importance of common species in maintaining ecosystem functioning^53^,^54^,^55^. Whilst the differences between alternative compensatory scenarios may be subtle, they can form crucial differences. Consequently, the repercussions of species loss are more effectively offset by a subset of species that share the same functional group or level of activity, especially at low levels of species richness. Whether these mechanisms can be identified and operate in natural communities, however, remains an open empirical question^56^.

It is important to acknowledge that our study is an abstraction of community dynamics and to recognize that the model assumptions we adopted represent an oversimplification of community interactions under environmental forcing. Our focus was not to predict the depth of sediment mixing for specific biodiversity futures, but rather to explore the relative importance of compensatory mechanisms in determining ecosystem properties. Whilst we were unable to incorporate the occurrence of co-extinctions^10^, non-indigenous invasive species^57^ or other cascading effects that can have further consequences for community structure and ecosystem functioning, we were able to establish divergent patterns in response for alternative extinction scenarios that hold promise for exploring new strategies of ecosystem management and governance. An important next step in predicting future biodiversity change, however, is to quantify the prevalence of local extinction drivers in the ecological landscape^58^ and understand how these interact in natural systems^59^,^60^ to influence the risk of extinction, altering community dynamics and ecosystem properties, both locally and across regional scales.

Extensive uncertainties exist in the responses of species and communities to environmental forcing, hence the use of empirically-based scenarios of the future to explore the potential consequences of species loss will continue to be a necessity for ecological advancements. We have shown, that the incorporation of important aspects of post-extinction community dynamics can lead to sharp contrasting forecasts of future ecosystem properties. Such information will help advance the predictability of community responses to change, provided that regionalised vulnerability assessments that determine the response of functionally important species under realistic future environmental conditions become available^61^,^62^. However, it is unlikely that all of these details will be available and incorporated into next generation models in the short-term, nor is this likely to be necessary. Consistency in community responses in the presence of compensation reveal patterns that may be general. Based on the available evidence, we should expect that the loss of species will be compensated by less efficient species over the long term^63^, resulting in alterations to ecosystem properties.

However, the discrepancy in ecosystem consequences between biomass and numeric compensatory responses emphasise the need to identify which, or whether both, compensatory processes prevail in natural assemblages. Estimates of the functional consequences of biodiversity loss that incorporate the error associated with such variation are needed, and will allow more confidence in simulations of the future and provide improved levels of certainty on the consequences of future global change.

## Methods

### Sampling and study site

Field data were collected at station Margaretta (22 m water depth, 53 ° 13.50′N, 09 ° 6.50′W) in Inner Galway Bay on the central west coast of Ireland. Samples of macro-invertebrates (retained on a 500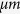 sieve; n = 5, 0.1 m^2^ van Veen grab) were collected approximately on a monthly basis over a one-year period (December 1996–November 1997, n = 11), returning a total of 139 invertebrate species^42^. Measurements of the sediment mixing depth were obtained using sediment profile imaging (SPI; n = 10) camera system^42^.

### Extinction simulations

Using a comprehensive study of the macrofaunal assemblages of Galway Bay, west coast of Ireland^42^, we predict how species extinction is likely to affect the mixing depth, an indicator of invertebrate bioturbation. We established the relationship between an index of bioturbation potential that uses per capita bioturbation potential (BP_i_, Supplementary Equation S1) to estimate population-level (BP_p_, Equation Supplementary S2) and community-level bioturbation potential (BP_c_, Supplementary Equation S3), which accounts for each species body size, abundance, activity level (4 levels, scored on a categorical scale reflecting increasing activity, 1 = in a fixed tube, 2 = limited movement, 3 = slow movement through sediment profile, 4 = free movement via burrow system^42^), and mode of sediment mixing (5 levels, scored on a categorical scale reflecting increasing impact on sediment turnover, 1 = epifauna, 2 = surfical modifiers, 3 = upward or downward conveyorbelt feeders, 4 = biodiffusers, 5 = regenerators^42^), and measurements of mixing depth obtained from sediment profile images. This relationship (Supplementary Equation S4) was used to parameterise probabilistic, numerical simulations that test how alternative extinction scenarios might affect sediment mixing depths. As environmental forcing in natural systems can target different components of the community^64^, we consider simulations in which species go extinct at random (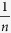, where n = the number of species) versus extirpations ordered by body size (largest expire first) or rarity (least abundant expire first). As the functional consequences of extinction also depend on the response of surviving species, we developed models in which species either do not exhibit compensatory responses or in which the abundance (numeric compensation) or biomass (biomass compensation) of the surviving community are held constant following extinction. In doing so, we recognized that compensating species are not randomly assigned, rather they represent different components of the species pool:

### Compensation by common species

Compensatory responses by common species is arguably one of the most likely pathways of compensation in a community, both probabilistically in terms of their relative proportion of abundance, and ecologically as numeric success reflects their disproportionate share of resources and competitive advantage over less numerous species^19^. In addition, this type of compensation has been observed in natural communities^52^.

### Compensation by rare species

The majority of community species are rare, but some species are equipped with unique functional traits^65^ and may become important if they increase in abundance^66^,^67^.

### Compensation by species from within/outwith or lower/higher functional grouping or which exhibit similar/distinct or lower/higher levels of activity

We assumed that species within the same functional group (e.g. sediment reworking mode^42^) will have similar functional traits and thus employ a similar ecological role, and that functional buffering can also be carried out by species from a different but adjacent functional group (e.g. grass versus forbs^19^, local versus non-local distribution of sediment particles by bioturbators^42^,^68^), or species selected from any lower or higher functional group. Compensating species can also exhibit similar or contrasting levels of activity (mobility categorisation^42^) relative to the species that has been extirpated, such that functional buffering can also be carried out by species from a different but adjacent mobility group, or species selected from any lower or higher mobility group. In the absence of an adjacent functional group, compensation stems from the next available functional group of greater/lesser or equal standing.

Each of these model scenarios (i.e. 3 extinction orders, 2 compensation mechanisms and 11 compensation types, n = 66) was run for 1000 simulations (from 139–1 species). We provide the code for executing each of these simulations in Supplementary Material (Code S1).

## Additional Information

**How to cite this article:** Thomsen, M. S. *et al*. Consequences of biodiversity loss diverge from expectation due to post-extinction compensatory responses. *Sci. Rep.* **7**, 43695; doi: 10.1038/srep43695 (2017).

**Publisher's note:** Springer Nature remains neutral with regard to jurisdictional claims in published maps and institutional affiliations.

## Supplementary Material

Supplementary Material
