# Supplementary material for: Consequences of biodiversity loss diverge from expectation due to post-extinction compensatory responses
Source: Sci Rep. 2017 Mar 3;7:43695. doi: 10.1038/srep43695 (PMC5334654; doi:10.1038/srep43695)
Supplement: Supplementary Material [file srep43695-s1.pdf]

Supplementary Material

Consequences of biodiversity loss diverge from expectation due to post-extinction compensatory responses

Authors: Matthias S. Thomsen[1*], Clement Garcia[2], Stefan G. Bolam[2], Ruth Parker[2] Jasmin A. Godbold[1,3] & Martin Solan[1].

[1]Ocean and Earth Science, National Oceanography Centre Southampton, University of Southampton Waterfront Campus, European Way, Southampton. SO14 3ZH, UK.

[2]Cefas Laboratory, Pakefield Road, Lowestoft, Suffolk, NR33 0HT, UK.

[3]Biological Sciences, University of Southampton, Highfield Campus, Southampton. SO17 1BJ, UK.

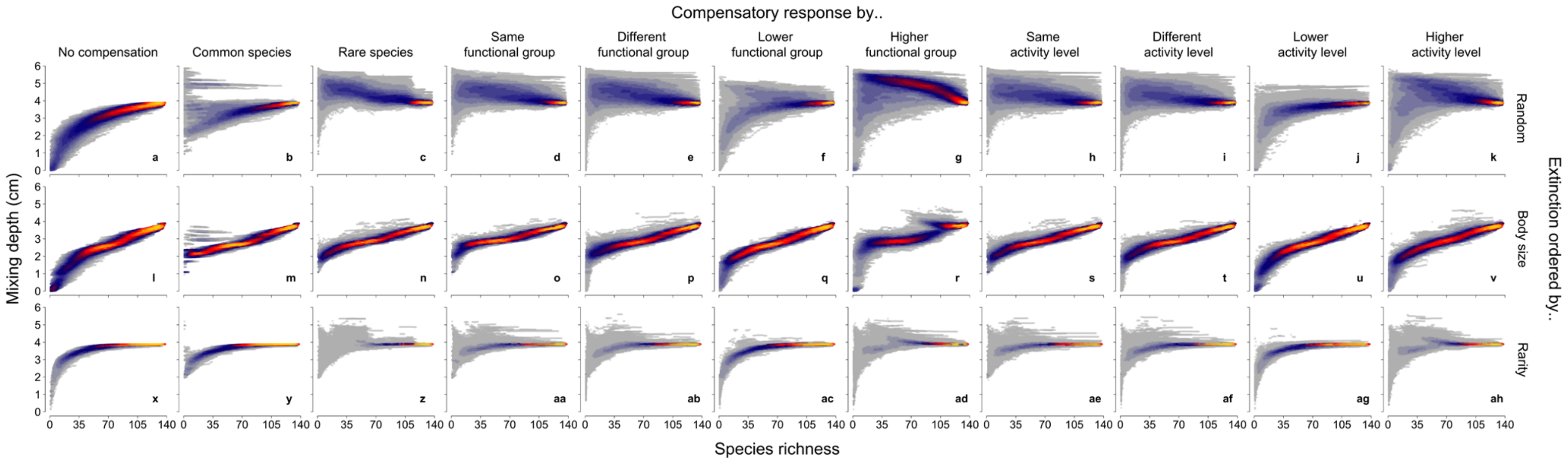

Figure S1| Predicted changes in sediment mixing depth following extinction of sediment-dwelling invertebrates and post-extinction numeric compensation without *Amphiura filiformis*. Simulations (n = 1000 per panel) represent species losses that occur at random (panels a-k) or which reflect trait-based vulnerabilities to extinction governed by body size (panels l-v) or rarity (panels w-ag). We assumed that the surviving community shows either no compensatory response (a, l, w) or full numeric compensation by common (b, m, x) or rare (c, n, r) species, species from within (d, o, z), between (e, p, aa), lower (f,q,ab) or higher (g,r,ac) functional groups, or species with the same (h, s, ad), different (i,t,ae), lower (j,u,af) or higher (k,v,ag) level of activity to the species that have gone extinct. Colour intensity (cold to warm colouration; grey - blue – red - yellow) reflects an increasing density (low to high) of data points.

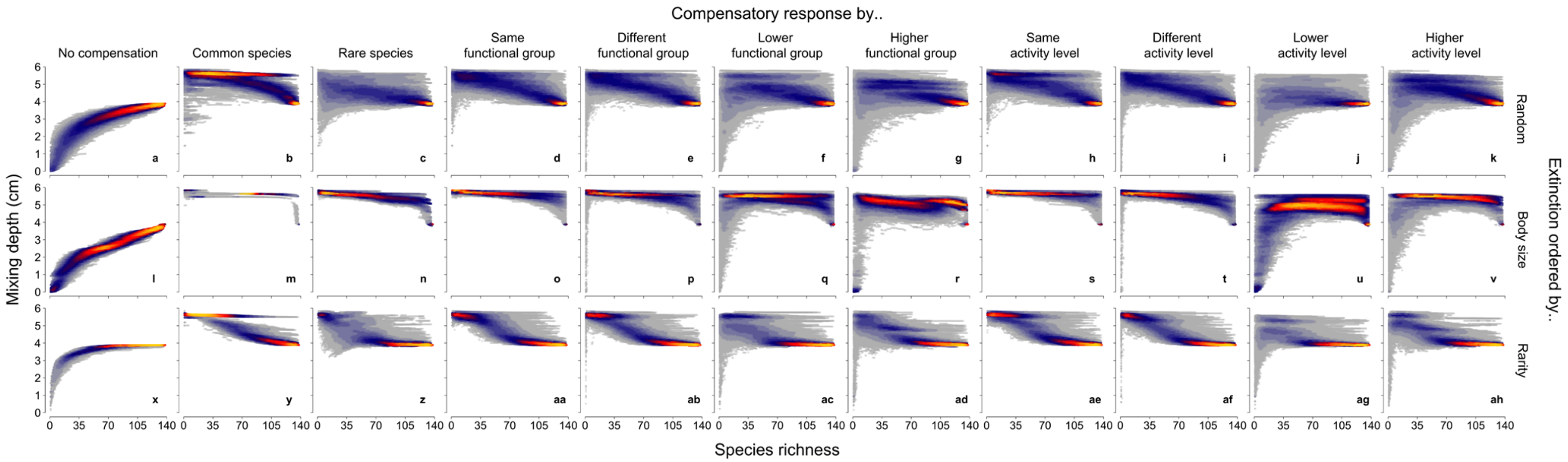

Figure S2| Predicted changes in sediment mixing depth following extinction of sediment-dwelling invertebrates and post-extinction biomass compensation without *Amphiura filiformis*. Simulations (n = 1000 per panel) represent species losses that occur at random (panels a-k) or which reflect trait-based vulnerabilities to extinction governed by body size (panels l-v) or rarity (panels w-ag). We assumed that the surviving community shows either no compensatory response (a, l, w) or full biomass compensation by common (b, m, x) or rare (c, n, r) species, species from within (d, o, z), between (e, p, aa), lower (f,q,ab) or higher (g,r,ac) functional groups, or species with the same (h, s, ad), different (i,t,ae), lower (j,u,af) or higher (k,v,ag) level of activity to the species that have gone extinct. Colour intensity (cold to warm colouration; grey - blue – red - yellow) reflects an increasing density (low to high) of data points.

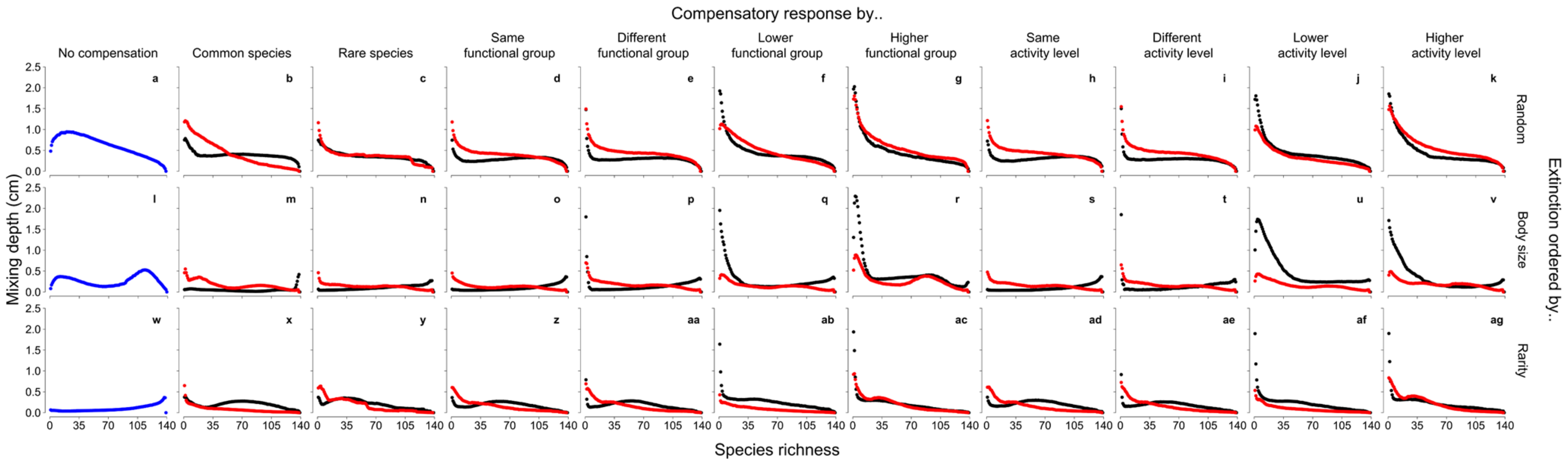

Figure S3 | Variability of sediment mixing depth following extinction of sediment-dwelling invertebrates and post-extinction compensation without *Amphiura filiformis*. Standard deviations are shown for the probabilistic distributions (n = 1000) at each level of species richness for the extinction scenarios and compensatory responses depicted in Figures 1 and 2, assuming no compensatory response (blue), full numeric compensation (red) or full biomass compensation (black).

Equation S1 | *Per capita* bioturbation potential, BP_i

$$BP_i = \overline{B}_i^{0.5} \times M_i \times R_i$$

B_i = mean species biomass (= total species biomass / total species abundance), in grams. Following Solan *et al.*[1], Bi is square root transformed to ensure species contributions conform to a linear scale.

R_i = a categorical scale to reflect increasing species effects on sediment turnover (1 = epifauna that bioturbate at the sediment-water interface; 2 = surficial modifiers, whose activities are restricted to <1-2 cm of the sediment profile; 3 = head-down/head-up feeders that actively transport sediment to/from the sediment surface; 4 = biodiffusers whose activities result in a constant and random diffusive transport of particles over short distances; and 5 = regenerators that excavate holes, transferring sediment at depth to the surface).

M_i = a categorical scale to reflect increasing activity of the species (1 = in a fixed tube; 2 = limited movement, sessile, but not in tube; 3 = slow movement through sediment; 4 = free movement via burrow system).

Equation S2 | Population level bioturbation potential, BP_p

$$BP_p = BP_i \times A_i$$

A_i = Mean total species abundance

Equation S3 | Community level bioturbation potential, BP_C

$$BP_c = \sum_{i=n}^{n} BP_P$$

Equation S4 | Mixing depth (MD).

The relationship between BP_c and the MD is based on an empirical fit[1].

$$MD = \left(\frac{6 \times e^{0.719 \times \log\left(BP_c\right) - 4.55}}{1 + e^{0.719 \times \log\left(BP_c\right) - 4.55}} \right)$$

Equation S4 places an upper bound on the mixing depth at zero (the sediment-water interface) and a lower bound at 6 cm (the lowest depth that animals typically burrow in Galway Bay[1]).

Code S1 | R code for Compensation Models: Worked Example

Our post-extinction compensation models can be implemented in the open source software R (https://www.r-project.org). Here, we provide a fully worked step-by step example of how to run our models on a typical faunal dataset. Note that there are certain prerequisites in terms of species information, data and format required to calculate the community bioturbation potential (BP_c), including individual species biomass, species population abundance, bioturbation mode and level of activity, for more detail see Solan *et al.*[1]. The code provided has been tested up to R version 3.3.0.

```
## Step 1. Get data in the correct format

## Data columns:
# - Species names
# - Mean species biomass (Bi)
# - Mean total species abundance (Ai)
# - Bioturbation potential per individual (BPi)
# - Bioturbation potential per population (BPp)
# - Sediment reworking mode (Ri)
# - Activity level (Mi)

# Save data as csv file.

## Step 2. Load data into R

# 1. Set working directory.

setwd("/Users/matthiasthomsen/Documents")

# 2. Load your data (csv) file.

alldata <- read.csv("Data_CompModels.csv", header=T)

# 3. Check data structure and that data has been loaded correctly.

str(alldata)
```

```
## 'data.frame':    139 obs. of  7 variables:
##  $ Species: Factor w/ 139 levels "Abra alba","Abra nitida",..: 1 2 3 4 5 6
7 8 9 10 ...
##  $ Ai     : num  0.7273 0.5454 2.8182 0.8182 0.0909 ...
##  $ Bi     : num  0.00071 0.00637 0.00051 0.00224 1.31381 ...
##  $ BPi    : num  0.107 0.319 0.09 0.379 2.292 ...
##  $ BPp    : num  0.0776 0.1741 0.2536 0.31 0.2084 ...
##  $ Ri     : int  2 2 2 4 1 4 4 4 2 3 ...
##  $ Mi     : int  2 2 2 2 2 3 3 3 1 2 ...

## Step 3.  Run simulation model
# The worked example below is for random extinction with no compensation.

# 1. For convenience store the number of species as a separate variable.

nsp <- nrow(alldata)

# 2. Set the number of simulations (in this case 100). This is the number of
times you wish to simulate species removal of the entire community (from
the total number of species until 1 species in the community remain).

nsims <- 100

# 3. Define whether compensation is present (TRUE) or absent (FALSE).

CompFlag <- FALSE

# 4. Add new columns (AiSim and EPSim) in the data file  (alldata) to record
the abundances and extinction probabilities for each simulation,
respectively.

alldata$AiSim <- NA
alldata$EPSim <- NA

# When compensation is driven by functional group or activity level, add two
new columns in the data file (alldata) to record functional group i.e.
reworking mode (Ri) and activity level (Mi), respectively.

alldata$RiSim <- NA # scenario: 5.C-D
alldata$MiSim <- NA # scenario: 5.E-F

# 5. Specify the probability of extinction, in this case random (all species
have equal risk of extinction, 1/nsp).

alldata$ExtinctProb <- 1/nsp

# To modify the scenario of extinction redefine the risk of extinction (i.e.
'alldata$ExtinctProb') by substituting '1/nsp' for the appropriate
probability of species extinction below.
```

```
# Extinction ordered by body size (largest to smallest):

# alldata$ExtinctProb <- alldata$Bi/sum(alldata$Bi)

# Extinction ordered by rarity (lowest to highest abundance):

# alldata$ExtinctProb <- (1/alldata$Ai)/sum(1/alldata$Ai)

# 6. Set up output data frame recording the simulated data. The columns are:

 # - Simulation: the simulation number.
 # - Nsp: the number of species remaining in the community
 # - ExtinctSpecies: the species that went extinct for a given simulation
run.
 # - Measure: the community function that is being calculated, in this case
the mixing depth (MD).
 # - Value: the value of that community function.
 # - CompensatingSpecies: the name of the compensating species for a given
simulation run.
 # - Compensation: the amount of compensation (varies with compensation type,
i.e. numeric or biomass).

# Note that the ExtinctSpecies column contains the name of the species that
will go extinct in the following run, i.e. the first row records the mixing
depth of the full, intact community, before any species has expired.

output <- expand.grid(Simulation = 1:nsims, Nsp = nsp:1, ExtinctSpecies=NA,
Measure=c("MD"), Value=NA, CompensatingSpecies=NA, Compensation=NA)

# 7. For clarity, order the Simulation column in the output data frame.

output <- output[order(output$Simulation),]

# 8. Having set up the output data frame for the model results, we can now
run the simulations.

for (sim_count in 1:nsims){
cat("sim_count: ", sim_count, "\n")

# 9. Reset abundances and the probability of extinction for the next
simulation.

alldata$AiSim <- alldata$Ai
alldata$EPSim <- alldata$ExtinctProb

# If compensation is present (i.e. CompFlag <- TRUE), reset the new for every
simulation run columns recording functional group (reworking mode) and
activity level.

alldata$RiSim <- alldata$Ri # scenario: 5.C-D)
```

```
alldata$MiSim <- alldata$Mi # scenario: 5.E-F)

# 10. Species removal from the total number of species to 1, knocking out
species according to probability of extinction.

for (sp_count in nsp:1)
{

# 11. Calculate the total community bioturbation potential (BPc).

BPc <- sum(alldata$AiSim * alldata$BPi)

# 12.Calculate the mixing depth of sediment particles by the faunal
community, based on an empirical fit¹ between BPc and the measured sediment
mixing depth (Equation S4).

MD <- (6*(exp((0.719*log(BPc))-4.55)))/(1+(exp((0.719*log(BPc))-4.55)))

# 13. Store these results in the output data frame.

output[output$Simulation == sim_count & output$Nsp==sp_count &
output$Measure=="MD", "Value"] <- MD

# 14. Random deletition of species based on the probability specified above
(see #8).

Extinct <- which(cumsum(alldata$EPSim)>=runif(1))[1]

# 15. If the capacity for compensation (i.e. “CompFlag”) is activated, the
following two functions (19-20) will be executed prior to removal of each
species, implementing compensation.

if(CompFlag==TRUE)
{

# 16. Calculate, as appropriate, the amount of biomass or abundance that will
be lost with the extirpated species, depending on the type of compensation
considered.

# 'BiomassLost' for compensatory responses by biomass.

BiomassLost <- alldata[Extinct,"AiSim"] * alldata[Extinct,"Bi"]

# OR 'abundanceLost' for numeric compensatory responses.

AbundanceLost <- alldata[Extinct,"AiSim"]

# 17. Specify the compensatory response by inserting the code (above the
closing bracket) for the appropriate scenario of compensation from step 5.
```

```r
}

# 18. Extinction happens. Set abundance and probability of extirpated species
to 0.

alldata[Extinct,c("EPSim","AiSim")] <- 0

# 19. Normalise the exinction probabilities so that the sum = 1.

alldata$EPSim <- alldata$EPSim / sum(alldata$EPSim)

# 20. Record the identity of the extipated species.

output[output$Simulation == sim_count & output$Nsp==sp_count,
"ExtinctSpecies"] <- as.character(alldata[Extinct,"Species"])
  }
}

## sim_count:  1
## sim_count:  2

...
## sim_count:  100

# Note that, if you are running multiple model scenarios after one another
you will have to adapt the code to accommodate for a unique id for each
output file corresponding to each model scenario.

## Step 4. Post-extinction compensation responses

# First, define compensation as present (see Step 3.3).

CompFlag <- TRUE

# SampleVar and isEmpty are required for the functional group and activity
level compensation scenarios.

# Function SampleVar allow the sampling of vectors of varying lengths (from x
components to 1).

SampleVar <- function(x) {
  if (length(x) <= 1) {
    return(x)
  } else {
    return(sample(x,1))
  }
}

# The function isEmpty is needed if there are no species left from the
selected functional group or activity level, in these rare instances,
compensation will instead be sustained by a species selected from a dif-
```

```
ferent functional group or activity level (depending on the appropriate com
pensation scenario) than originally selected for.

isEmpty <- function(x) {
  return(length(x)==0)
}

## Compensatory responses by:
# 4.A: Common species
# 4.B: Rare species
# 4.C: Same functional group
# 4.D: Different functional group
# 4.E: Lower functional group
# 4.F: Higher functional group
# 4.G: Same activity level
# 4.H: Different activity level
# 4.I: Lower activity level
# 4.J: Higher activity level
# 4.K: Record amount of compensation

# Insert code for the appropriate compensatory response in step 3.20 (within
the curly brackets).

## 4.A Common species compensate

# Removes the species extirpated for a given simulation.

temp<-alldata[-c(Extinct),]

# Removes all the species gone extinct previously in the simulation (AiSim =
0).

temp<-temp[!temp$AiSim %in% 0,]

# Order species remaining in the community according to abundance (highest to
lowest).

HighestAbn<-as.numeric(rownames(temp[order(temp$AiSim, decreasing=T),][1,]))

# The most abundant species compensates, as appropriate, by the biomass or
abundance lost with extirpated species.

# Biomass compensation.

alldata$AiSim[HighestAbn] <- alldata$AiSim[HighestAbn] +
BiomassLost/alldata$Bi[HighestAbn]

# OR numeric compensation.

alldata$AiSim[HighestAbn] <- alldata[Extinct,"AiSim"] +
```

```
alldata$AiSim[HighestAbn]

# Record the identity of the species with compensating capacity in the output
file.

output[output$Simulation == sim_count &
output$Nsp==sp_count,"CompensatingSpecies"] <-
as.character(alldata$Species[HighestAbn])



## 4.B Rare species compensate

# The "-Extinct" is to tell R to take all abundance value but the one from
the extinct species and order them by their row number.

# Removes the species extirpated for a given simulation.

temp <- alldata[-c(Extinct),]

# Removes all the species gone extinct previously in the simulation (AiSim =
0).

temp <- temp[!temp$AiSim %in% 0,]

# Order species remaining in the community at any given time of the
simulation according to abundance (lowest to highest).

LowestAbn <- as.numeric(row.names(temp[order(temp$AiSim),][1,]))

# The species of lowest abundance compensates, as appropriate, by the biomass
or abundance lost with the extirpated species.

# Biomass compensation.

alldata$AiSim[LowestAbn] <- alldata$AiSim[LowestAbn] +
BiomassLost/alldata$Bi[LowestAbn]

# OR numeric compensation.

alldata$AiSim[LowestAbn] <- alldata[Extinct,"AiSim"] +
alldata$AiSim[LowestAbn]

# Record the identity of the species with compensating capacity in the output
file.

output[output$Simulation == sim_count &
output$Nsp==sp_count,"CompensatingSpecies"] <-
as.character(alldata$Species[LowestAbn])
```

```
## 4.C Same functional group compensation

# Calculate the numerical distance in terms of reworking mode for all
remaining species in the community in reference to species selected for
extinction.

distance<-dist(alldata$RiSim, diag = TRUE, upper = TRUE)

# Create distance matrix.

distance<-as.matrix(distance)

diag(distance) <- NA

# Define vector of species distances in reworking mode to the extinct
species, excluding the distance of the extirpated species with itself.

d1D<-distance[,Extinct]

# Create vector d1D containing the distance for each species remaining in the
community to the extinct species. Select species with most similar
reworking trait (i.e. shortest distance to extirpated species), omitting
species that   have already gone extinction previously in the simulation
(i.e. NA's).

d1D<-d1D[d1D==min(d1D, na.rm=TRUE)]

# Remove species that are already gone extinct previously in the simulation
(i.e. NA) from vector d1D.

d1D<-d1D[!is.na(d1D)]

# Select one species from vector d1D.

RdmRi<-SampleVar(names(d1D))

# Translate back into numeric.

RdmRi<-as.numeric(RdmRi)

# Species of similar reworking trait compensates, as appropriate, by the
biomass or abundance lost with the extirpated species.

# Biomass compensation.

alldata[RdmRi, "AiSim"] <- alldata[RdmRi, "AiSim"] +
BiomassLost/alldata[RdmRi, "Bi"]

# Numeric compensation.
```

```r
alldata[RdmRi, "AiSim"] <- alldata[Extinct,"AiSim"] + alldata[RdmRi, "AiSim"]

# Record the identity of the species with compensating capacity in the output file.

output[output$Simulation == sim_count &
output$Nsp==sp_count,"CompensatingSpecies"] <- ifelse(!isEmpty(RdmRi),
as.character(alldata$Species[RdmRi]), NA)


## 4.D Between functional group compensation

# Calculate the numerical distance in terms of reworking mode for all remaining species in the community in reference to species selected for extinction.

distance<-dist(alldata$RiSim, diag = TRUE, upper = TRUE)

# Create distance matrix

distance<-as.matrix(distance)

diag(distance) <- NA

# Define vector of species distances in reworking mode to the extinct species, excluding the distance of the extirpated species with itself.

d1D<-distance[,Extinct]

# Create vector d1D containing the species remaining in the community excluding all species with the same reworking trait to that of the extinct species. Select species with different activity level.

d1D<-d1D[!d1D %in% 0]

# Remove species that are already gone extinct (i.e. NA’s) from vector d1D.
d1D<-d1D[!is.na(d1D)]

# Select one species from vector d1D.

RdmRi<-SampleVar(names(d1D))

# Translate back into numeric.

RdmRi<-as.numeric(RdmRi)

# Species of dissimilar reworking trait compensates, as appropriate, by the biomass or abundance lost with the extirpated species.
```

```
# Biomass compensation.

alldata$AiSim[RdmRi] <- alldata$AiSim[RdmRi] + BiomassLost/alldata$Bi[RdmRi]

# OR numeric compensation.

alldata$AiSim[RdmRi] <- alldata[Extinct,"AiSim"] + alldata$AiSim[RdmRi]

# Record the identity of the compensating species in the output file.

output[output$Simulation == sim_count &
output$Nsp==sp_count,"CompensatingSpecies"] <- ifelse(!isEmpty(RdmRi),
as.character(alldata$Species[RdmRi]), NA)



## 4.E Lower functional group

# Determine the functional (reworking, Ri) group of the extinct species.

RiExtinct <- alldata[Extinct,"Ri"]

# Create temporary data file (TEMPalldata) to avaoid NAs.

TEMPalldata<-alldata[complete.cases(alldata),]

# Select species with capacity for compensation by nesting two ifelse
functions.

# Condition statement: Are there any species in alldata$RiSim from a lower
functional group than the species that went extinct (i.e. RiExtinct).

LowRi<-ifelse(length(TEMPalldata$RiSim[TEMPalldata$RiSim < RiExtinct]) > 0,

# If yes, the SampleVar function selects one element from this group.

SampleVar(rownames(TEMPalldata[TEMPalldata$RiSim < RiExtinct,])),

# If no, are there any species in the remaining community (i.e.
alldata$RiSim) from the same fucntional group to that of the species that
went extinct (i.e. RiExtinct).

ifelse(length(TEMPalldata$RiSim[TEMPalldata$RiSim == RiExtinct]) > 0,

# If yes, the SampleVar function selects one element from this group.

SampleVar(rownames(TEMPalldata[TEMPalldata$RiSim == RiExtinct,])),

# If no, the SampleVar function selects one element from a functional group
higher than that of the species that went extinct (i.e. RiExtinct).
```

```
SampleVar(rownames(TEMPalldata[TEMPalldata$RiSim > RiExtinct,]))))

# Translate back into numeric.

LowRi<-as.numeric(LowRi)

# Now add compensation by either biomass OR numeric.

# Add biomass of the species lost to the compensating species (from a lower
functional group).

alldata$AiSim[LowRi] <- alldata$AiSim[LowRi] + BiomassLost/alldata$Bi[LowRi]

# Record ID of the compensating species.

outputB[outputB$Simulation == sim_count &
outputB$Nsp==sp_count,"CompensateSpecies"] <- ifelse(!isEmpty(LowRi),
as.character(alldata$Species[LowRi]), NA)


## 4.F Higher functional group

# Determine the functional (reworking, Ri) group of the extinct species.

RiExtinct <- alldata[Extinct,"Ri"]

# create temporary data file (TEMPalldata) to avaoid NAs.

TEMPalldata<-alldata[complete.cases(alldata),]

# Select species with capacity for compensation by nesting two ifelse
functions.

# Condition statement: Are there any species in alldata$RiSim from a higher
functional group than the species that went extinct (i.e. RiExtinct).

HighRi<-ifelse(length(TEMPalldata$RiSim[TEMPalldata$RiSim > RiExtinct]) > 0,

# If yes, the SampleVar function selects one element from this group.

SampleVar(rownames(TEMPalldata[TEMPalldata$RiSim > RiExtinct,])),

# If no, are there any species in the remaining community (i.e.
alldata$RiSim) from the same fucntional group to that of the species that
went extinct (i.e. RiExtinct).

ifelse(length(TEMPalldata$RiSim[TEMPalldata$RiSim == RiExtinct]) > 0,
```

```r
# If yes, the SampleVar function selects one element from this group.

SampleVar(rownames(TEMPalldata[TEMPalldata$RiSim == RiExtinct,])),

# If no, the SampleVar function selects one element from species from a
functional group that is lower than that of the species that went extinct
(i.e. RiExtinct).

SampleVar(rownames(TEMPalldata[TEMPalldata$RiSim < RiExtinct,]))))

# Translate back into numeric.

HighRi<-as.numeric(HighRi)

# Now add compensation by either biomass OR numeric.

# Add biomass of the species lost to species from a higher functional group.

alldata$AiSim[HighRi] <- alldata$AiSim[HighRi] +
BiomassLost/alldata$Bi[HighRi]

# Record ID of the compensating species.

outputA[outputA$Simulation == sim_count &
outputA$Nsp==sp_count,"CompensateSpecies"] <- ifelse(!isEmpty(HighRi),
as.character(alldata$Species[HighRi]), NA)


## 4.G Same activity level compensate

# Calculate the numerical distance in terms of activity level for all
remaining species in the community in reference to species selected for
extinction.

distance<-dist(alldata$MiSim, diag = TRUE, upper = TRUE)

# Create distance matrix.

distance<-as.matrix(distance)

diag(distance) <- NA

# Define vector of species distances in activity level to the extinct
species, excluding the distance of the extirpated species with itself.

d1D<-distance[Extinct,]

# Create vector d1D containing the distance for each species remaining in the
community to the extinct species. Select species with most similar activity
```

```r
level (i.e. shortest distance to extirpated species), omitting species that
have already gone extinction (i.e. NA's).

d1D<-d1D[d1D==min(d1D, na.rm=TRUE)]

# Remove species that are already gone extinct (i.e. NA) from vector d1D.

d1D<-d1D[!is.na(d1D)]

# Select one species from vector d1D.

RdmMi<-SampleVar(names(d1D))

# Translate back into numeric.

RdmMi<-as.numeric(RdmMi)

# Species of same level of activity compensates, as appropriate, by the
biomass or abundance lost with the extirpated species.

# Biomass compensation.

alldata[RdmMi, "AiSim"] <- alldata[RdmMi, "AiSim"] +
BiomassLost/alldata[RdmMi, "Bi"]

# OR numeric compensation.

alldata[RdmMi, "AiSim"] <- alldata[Extinct,"AiSim"] + alldata[RdmMi, "AiSim"]

# Record the identity of the species with compensating capacity in the output
file.

output[output$Simulation == sim_count &
output$Nsp==sp_count,"CompensatingSpecies"] <- ifelse(!isEmpty(RdmRi),
as.character(alldata$Species[RdmRi]), NA)


## 4.H Different activity level compensate

# Calculate the numerical distance in terms of activity level for all
remaining species in the community in reference to species selected for
extinction.

distance<-dist(alldata$MiSim, diag = TRUE, upper = TRUE)

# Create distance matrix.

distance<-as.matrix(distance)

diag(distance) <- NA
```

```r
# Define vector of species distances in activity level to the extinct species, excluding the distance of the extirpated species with itself.

d1D<-distance[Extinct,]

# Create vector d1D containing the species remaining in the community excluding all species from the same activity level of the extinct species. Select species with different activity level.

d1D<-d1D[!d1D %in% 0]

# Remove species that are already gone extinct (i.e. NA’s) from vector d1D.

d1D<-d1D[!is.na(d1D)]

# Select one species from the d1D vector.

RdmMi<-SampleVar(names(d1D))

# Translate back into numeric.

RdmMi<-as.numeric(RdmMi)

# Species of different activity level compensates, as appropriate, by the biomass or abundance lost with the extirpated species.

# Biomass compensation.

BiomassLost/alldata$Bi[RdmMi]
alldata[RdmMi, "AiSim"] <- alldata[RdmMi, "AiSim"] + BiomassLost/alldata[RdmMi, "Bi"]

# OR numeric compensation.

alldata$AiSim[RdmMi] <- alldata[Extinct,"AiSim"] + alldata$AiSim[RdmMi]

# Record the identity of the species with compensating capacity in the output file.

output[output$Simulation == sim_count & output$Nsp==sp_count,"CompensatingSpecies"] <- ifelse(!isEmpty(RdmRi), as.character(alldata$Species[RdmRi]), NA)


## 4.I Lower activity level

# Determine the acitivity level (mobility mode, Mi) of the extinct species.

MiExtinct <- alldata[Extinct,"Mi"]
```

```
# create temporary data file to avoid NAs.

TEMPalldata<-alldata[complete.cases(alldata),]

# Select species with capacity for compensation by nesting two ifelse
functions.

# Condition statement: If there are any species in alldata$MiSim from a lower
functional group to the species went extinct (i.e. MiExtinct).

LowMi<-ifelse(length(TEMPalldata$MiSim[TEMPalldata$MiSim < MiExtinct]) > 0,

# If yes, the SampleVar function selects one element from this group.

SampleVar(rownames(TEMPalldata[TEMPalldata$MiSim < MiExtinct,])),

# If no, are there any element of alldata$MiSim of the same activity level to
that of the species that went extinct (i.e. MiExtinct).

ifelse(length(TEMPalldata$MiSim[TEMPalldata$MiSim == MiExtinct]) > 0,

# If yes, the SampleVar function selects one element from this group.

SampleVar(rownames(TEMPalldata[TEMPalldata$MiSim == MiExtinct,])),

# If no, the SampleVar function selects one species from an activity level
higher than the species that went extinct.

SampleVar(rownames(TEMPalldata[TEMPalldata$MiSim > MiExtinct,]))))

# Translate back into numeric.

LowMi<-as.numeric(LowMi)

# Now add compensation by either biomass or numeric.

# Add biomass from the species lost to compensating species (from a lower
functional group).

alldata$AiSim[LowMi] <- alldata$AiSim[LowMi] + BiomassLost/alldata$Bi[LowMi]

# Record ID of who has compensated.

outputD[outputD$Simulation == sim_count &
outputD$Nsp==sp_count,"CompensateSpecies"] <-     ifelse(!isEmpty(LowMi),
as.character(alldata$Species[LowMi]), NA)


## 4.J Higher activity level
```

```
# Determine the acitivity level (mobility mode, Mi) of the extinct species.

MiExtinct <- alldata[Extinct,"Mi"]

# create temporary data file to avaoid NAs.

TEMPalldata<-alldata[complete.cases(alldata),]

# Select species with capacity for compensation by nesting two ifelse
  functions.

# Condition statement: Are there any species in alldata$MiSim from a higher
activity level (Mi)  than the species that went extinct (i.e. MiExtinct).

HighMi<-ifelse(length(TEMPalldata$MiSim[TEMPalldata$MiSim > MiExtinct]) > 0,

# If yes, the SampleVar function selects one element from this group.

SampleVar(rownames(TEMPalldata[TEMPalldata$MiSim > MiExtinct,])),

# If no, are there any element of alldata$MiSim of the same activity level to
that of the species that went extinct (i.e. MiExtinct).

ifelse(length(TEMPalldata$MiSim[TEMPalldata$MiSim == MiExtinct]) > 0,

# If yes, the SampleVar function selects one element from this group.

SampleVar(rownames(TEMPalldata[TEMPalldata$MiSim == MiExtinct,])),

# If no, the SampleVar function selects one species from an activity level
lower than the species that went extinct.

SampleVar(rownames(TEMPalldata[TEMPalldata$MiSim < MiExtinct,]))))

# Translate into numeric.

HighMi<-as.numeric(HighMi)

# Now add compensation by either biomass OR numeric.

# Add biomass of the species lost to species with higher activity level.

alldata$AiSim[HighMi] <- alldata$AiSim[HighMi] +
BiomassLost/alldata$Bi[HighMi]

# Record ID of the compensating species.

outputC[outputC$Simulation == sim_count &
```

```
outputC$Nsp==sp_count,"CompensateSpecies"] <- ifelse(!isEmpty(HighMi),
as.character(alldata$Species[HighMi]), NA)


## 4.K Record amount of compensation

# Record the amount of biomass or numeric compensation, as appropriate, in
output file. Note that this line of code needs to be inserted after the
command line recording the compensating species, but within the closing
bracket in step 3.20.

# Record biomass compensation.

output[output$Simulation == sim_count &
output$Nsp==sp_count,"BiomassCompensation"] <-BiomassLost

# OR record numeric compensation.

output[output$Simulation == sim_count & output$Nsp==sp_count,"Compensation"]
<- alldata[Extinct,"AiSim"]
```

```
## Step 5. Plot model results

# Plot model output using the heatscatter command. First, install the LSD
package available at the CRAN repository. Once installed, load the LSD
package into your library.

# Install LSD package by removing the hash for the line below, and run code.
# install.packages("LSD").

# Load LSD packaged.
library(LSD)

par(mar = c(6,6,1,1))

heatscatter(output$Nsp,output$Value,
            bty="n",
            pch = 19,
            cexplot = 1,
            nrcol = 30,
            grid = 200,
            colpal = "heat",
            xlab = "",
            ylab = "",
            xaxt = "n",
            main = "",
            las = 1,
            cex.lab = 1.6,
```

```
            cex.axis = 1.6,
            ylim = c(0.0,6.0),
            xlim = c(0.0,139.5),
            alpha = 50)
axis(1, at = seq(0,140, by = 35), lab = c("0","35","70","105","140"),
     las = 1, cex.lab = 1.6, cex.axis = 1.6, mgp = c(0,1,0))
title(xlab = "Species richness", ylab = "Mixing depth (cm)", cex.lab = 2)
```

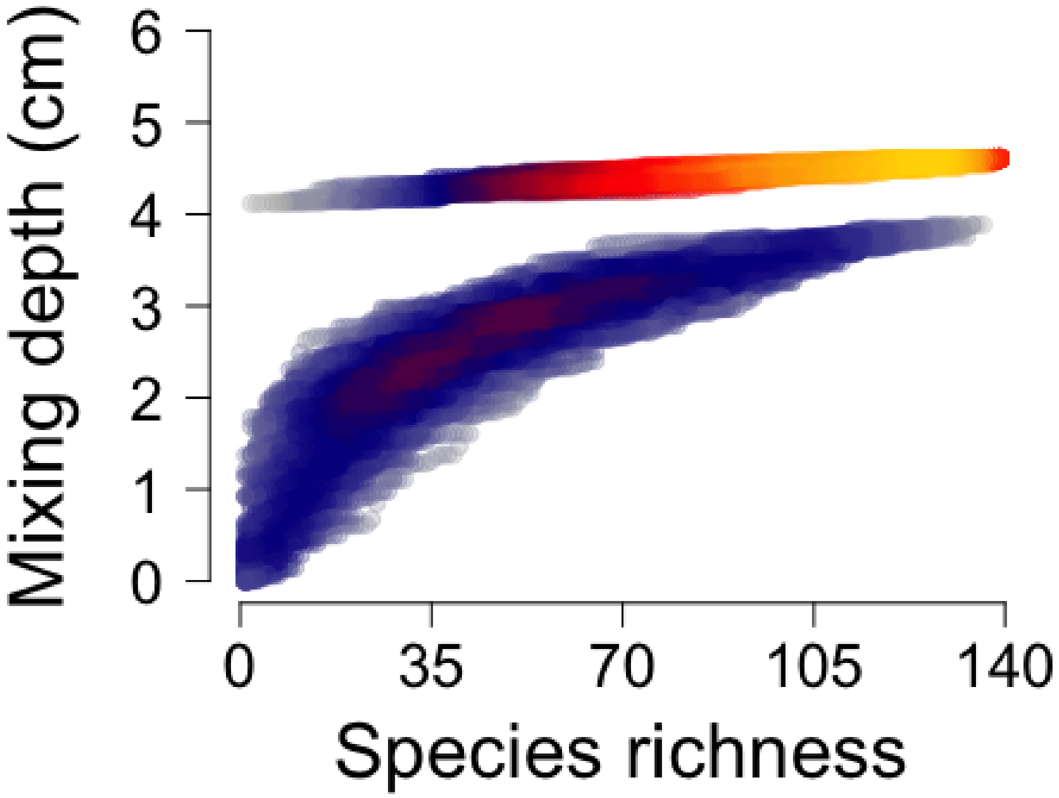


```
# Step 6. Save results to csv file.

# Use the write function to save data output as a csv file.

# Mac users:
write.csv(output, "~/Desktop/output.csv", row.names=T)

# Windows users:
write.csv(output, "C:/ Users/UserName/Desktop/output.csv", row.names=T)
```

References

1. Solan, M. *et al*. Extinction and ecosystem function in the marine benthos. *Science* **306**, 1177-1180 (2004).
